# Acute Albuminuria of Pregnancy

**Published:** 1876-01

**Authors:** 


Acute Albuminuria of Pregnancy.—Dr. Richardson reported the case of a primipara twenty-four years old, who had entered the Boston Lying-in Hospital three weeks previously, and two weeks before her confinement. At the time of her entrance the urine was found free from albumen, and otherwise perfectly normal. The labor began in the evening. At this time the patient passed her water, which was found to contain a slight amount of albumen. At four A.M. next day she could not micturate, and four or five ounces of urine were drawn by the catheter, and found to contain a large amount of albumen, together with epithelial casts. At 7:30 the urine was very scanty, only about an ounce and a half. It was loaded with albumen and contained very numerous casts. The patient, who had previously shown a good disposition, now became ugly and cross. She was drowsy and restless, and complained of terrible headache. The catheter drew less than half an ounce of urine. At eleven A.M. the forceps were applied. At four P.M. six or seven ounces of urine were passed. This contained a large amount of albumen, but the casts were much diminished. The next morning, twenty-four hours after delivery, ten ounces of urine were obtained, which showed simply a trace of albumen. That afternoon the urine was wholly free from both albumen and casts. The patient had not had a bad symptom since, and was now making a good recovery. The specific gravity of the urine varied from 1018 to 1024. There was very slight œdema of the feet. When the forceps were applied the head had passed the superior strait and had entered the pelvis. The os was two-thirds dilated, and was very soft and dilatable. The labor had been going on very gradually and slowly till about seven or eight A.M., and from that time the pains seemed to die away. The patient was etherized for the operation. The placenta was adherent at one spot, over a surface as large as a silver half-dollar, and all around the site of adhesion were thin plates of calcareous degeneration.—*Ibid.*

